# A 12-month randomised pilot trial of the Alzheimer’s and music therapy study: a feasibility assessment of music therapy and physical activity in patients with mild-to-moderate Alzheimer’s disease

**DOI:** 10.1186/s40814-023-01287-1

**Published:** 2023-04-19

**Authors:** A. M. Matziorinis, B. K. Flo, S. Skouras, K. Dahle, A. Henriksen, F. Hausmann, T. T. Sudmann, C. Gold, S. Koelsch

**Affiliations:** 1 Department of Biological and Medical Psychology, University of Bergen, Bergen, Norway; 2 Kompetansesenter for Demens, Bergen Kommune, Norway; 3 Department of Health and Function, Western Norway University of Applied Sciences, Bergen, Norway; 4 Department of Sport, Food, and Natural Sciences, Faculty of Education, Arts, and Sports, Western Norway University of Applied Sciences, Bergen, Norway; 5 NORCE Norwegian Research Centre AS, Bergen, Norway; 6 Grieg Academy Department of Music, University of Bergen, Bergen, Norway; 7 Department of Clinical and Health Psychology, University of Vienna, Vienna, Austria

**Keywords:** Non-pharmacological therapies, Music therapy, Physical activity, Randomised pilot trial, Feasibility, Alzheimer’s disease, Longitudinal, Randomised controlled trial

## Abstract

**Background:**

The Alzheimer’s and Music Therapy (ALMUTH) study is the first randomised controlled trial (RCT) design with 12 months of active non-pharmacological therapy (NPT) implementing music therapy (MT) and physical activity (PA) for participants with Alzheimer’s disease (AD). The aim of the present article is to retrospectively examine the inclusion of mild-to-moderate Alzheimer’s Disease patients into the main ALMUTH study protocol and to determine if continued inclusion of AD patients is warranted.

**Methods:**

The randomised pilot trial was conducted as a parallel three-arm RCT, reflecting the experimental design of the ALMUTH study. The trial was conducted in Bergen, Norway, and randomisation (1:1:1) was performed by an external researcher. The study was open label and the experimental design features two active NPTs: MT and PA, and a passive control (no intervention, CON) in Norwegian speaking patients with AD who still live at home and could provide informed consent. Sessions were offered once per week (up to 90 min) up to 40 sessions over 12 months. Baseline and follow-up tests included a full neuropsychological test battery and three magnetic resonance imaging (MRI) measurements (structural, functional, and diffusion weighted imaging). Feasibility outcomes were assessed and were determined as feasible if they met the target criteria.

**Results:**

Eighteen participants with a diagnosis of mild-to-moderate AD were screened, randomised, and tested once at baseline and once after 12-months. Participants were divided into three groups: MT (*n* = 6), PA (*n* = 6), and CON (*n* = 6). Results of the study revealed that the ALMUTH protocol in patients with AD was not feasible. The adherence to the study protocol was poor (50% attended sessions), with attrition and retention rates at 50%. The recruitment was costly and there were difficulties acquiring participants who met the inclusion criteria. Issues with study fidelity and problems raised by staff were taken into consideration for the updated study protocol. No adverse events were reported by the patients or their caregivers.

**Conclusions:**

The pilot trial was not deemed feasible in patients with mild-to-moderate AD. To mitigate this, the ALMUTH study has expanded the recruitment criteria to include participants with milder forms of memory impairment (pre-AD) in addition to expanding the neuropsychological test battery. The ALMUTH study is currently ongoing through 2023.

**Trial registration:**

Norsk Forskningsråd (NFR) funded. Regional Committees for Medical and Health Research Ethics (REC-WEST: reference number 2018/206). ClinicalTrials.gov: NCT03444181 (registered retrospectively 23 February 2018, https://clinicaltrials.gov/ct2/show/NCT03444181).

## Key messages on feasibility

- What uncertainties about feasibility existed prior to this study?

To our knowledge there are currently no other 12-month RCT neuroimaging studies implementing non-pharmacological therapies in mild-to-moderate AD patients, thus it was uncertain how patients would react to the demands of the ALMUTH study protocol. It is also the only study to our knowledge that evaluates neuroscientific methods to investigate the effects of NPTs such as MT and PA on brain plasticity and brain ageing in Alzheimer patients. Since there are uncertainties regarding recruitment, testing, attrition, retention, and adherence of patients to such an intervention, it was imperative to ensure whether the inclusion of mild-to-moderate AD patients was feasible and whether the continuation of recruitment of AD individuals was warranted in the main study.

- What are the key feasibility findings from this study?

Key feasibility findings of the study revealed that the participants had difficulty adhering to the protocol. Due to acceleration of AD symptoms and disease progression, half of the included patients withdrew from the study leading to an attrition rate of 50% (9/18) and retention rates 50% (9/18). Of those who completed a post-assessment 12-months later, adherence to protocol was 50% with many issues raised by staff for further change or upgrades to the main study protocol. These findings prompted the ALMUTH study to expand the recruitment criteria to pre-AD stages (such as Mild Cognitive Impairment and Subjective Cognitive Decline), and to adapt the study protocol [1].

- What are the implications of the feasibility findings on the main study's design?

Generally regarded as good practice, the publication of feasibility studies, particularly in phase III trials including randomisation, is very instructive for future studies on Alzheimer’s Disease. Implications of the randomised pilot trial revealed that the feasibility of conducting such an intensive, weekly intervention over the course of 12-months, including baseline and follow-up neuropsychological, and neuroscientific assessments in Alzheimer patients was not feasible in our sample. These findings allowed the ALMUTH study to expand inclusion criteria to include participants with a diagnosis of Mild Cognitive Impairment and participants with subjective memory changes who met the criteria for Subjective Memory Decline. The impetus for their inclusion is based on research that Alzheimer’s disease begins decades before the clinical presentation of AD symptoms [2, 3] and that the preventative use of NPTs in presymptomatic AD stages may ameliorate disease outcomes.

## Background

As the most widespread and fatal progressive neurodegenerative disorder among the elderly, the prevalence of Alzheimer’s Disease has been accelerating, making up 60–80% of all dementia cases worldwide [4]. Currently, there are 55 million people worldwide living with dementia, and its incidence is estimated to double every 20 years [5]. The pathological development of AD has been recognised to exist on a continuum, known as the Alzheime's continuum, with the disease beginning in a preclinical phase and developing towards prodromal AD (also known as amnestic Mild Cognitive Impairment (aMCI) due to AD), and finally expressed as a clinical syndrome of AD dementia with increasing severity including impairing activities of daily living (ADL) [4].

The defining pathological features characterising AD are a spatiotemporal spread of amyloid beta (Aβ) protein in senile plaques, tau protein neurofibrillary tangles, neurodegeneration, and vascular amyloidopathy [6]. Clinical and cognitive deficits of AD include memory loss that disrupts daily life, challenges in executive functioning, planning, problem solving, trouble understanding images and spatial relationships, changes in mood and personality, withdrawal from work and social activities, decreased judgment, and novel difficulties with words in speech and/or writing [7].

As the efficacy of drug interventions for AD has not yet proven to be satisfactory [8–10], NPTs such as physical exercise, music therapy, light therapy, cognitive behavioural therapy, and diet to prevent and relieve symptoms of AD have been implemented alongside pharmaceutical interventions [11–13]⁠. Due to their low cost ⁠[14] and relatively easy implementation, NPTs are recommended as adjunctive therapies, preferably at the earliest opportunity [15], in early and presymptomatic AD stages [16]⁠. The increased integration and use of NPTs in conjunction with approved drug therapies may aid in diminishing the percentage of preclinical and prodromal individuals converting to AD.

### Music therapy in AD

Music therapy has shown promise as a non-pharmacological therapy for Alzheimer’s disease [17, 18]. Several studies have shown spared musical memory in patients with AD [19–21], and is evidenced by their ability to recognise familiar music [22]⁠. Music activates a broad network in the brain, rather than being localised to a specific area [23, 24]⁠. Brain areas underlying musical memory are among the last to show brain atrophy in AD [25]⁠, and thus the implementation of music in a medical setting has been encouraged [26]. Moreover, music has been shown to reduce anxiety in patients with AD [27, 28], improve cognitive performance in tasks related to verbal and episodic memory, enhance the encoding and retrieval of verbal information [29, 30], and improve autobiographical recall [31–33].⁠⁠

Music therapy can contribute to improvements in cognition and neuropsychiatric symptoms of AD [34, 35], and decreasing symptoms related to agitation, depression, anxiety, and overall behavioural symptoms [28, 36–38]⁠. Another study highlighted that music therapy can increase happiness scores, lower stress levels, and improve emotional state in patients with mild AD [39]⁠. A functional magnetic resonance imaging (fMRI) study conducted by Satoh and colleagues [40]⁠ using singing training led to improvements in neuropsychiatric symptoms as well as neural efficacy of cognitive processing in patients with AD. Lyu and colleagues explored the effects of music therapy (singing) on cognitive function and mental well-being of patients with varying severity of AD and found that MT was more effective for improving verbal fluency and alleviating psychiatric symptoms and caregiver distress compared to a lyric reading condition [41]. Although effects subsided after 3 months, the authors suggest that group music interventions can be effective in improving social interaction between people with dementia. Further research on singing has also shown positive effects on heart rate variability [42]⁠ and social bonding [43]⁠, and can improve memory, mood, and the relationship to a caregiver [37]⁠.

Moreover, music therapy can improve attention, psychomotor speed, memory, orientation, and executive functions [40, 41] Meta-analyses [44, 45] have indicated that music therapy has positive effects in AD patients, including improvements of cognitive function, global cognition, quality of life, and ameliorating symptoms of long-term depression [46]. However, the relative methodological quality of such studies was low, resulting in weak effect sizes, and a lack of longitudinal studies demonstrating long-term benefits [18, 47]. A meta-analysis of 15 studies by Wang, Zhan, and Cai [48] showed that the effects of MT on cognitive function and activities of daily living (ADL) scores were not significant in patients with AD. A systematic review and meta-analysis by Fusar-Poli and colleagues [45] analysed 110 studies on the effect of MT on cognitive functions in patients with dementia and found that although there were beneficial effects on global cognition, RCT studies with larger sample sizes will be required to elucidate the impact of MT on cognitive functions in patients with dementia. Thus, there is still a paucity of solid scientific evidence for positive effects of MT on cognitive function and brain degeneration in AD.

### Physical activity/exercise therapy in AD

Physical activity has been implicated as a potential NPT that can help reduce the incidence of dementia and AD. As an estimated 54% of risk factors for developing AD may be preventable [49]⁠, it was found that the highest attributable risk factor for developing AD was physical inactivity [50]⁠. A literature review by Cass [51] reviewed exercise treatments and AD revealing that exercise can improve brain blood flow, increase hippocampal volume and cognitive performance [52]. A study which analysed the effect of over a year of mild to moderate physical activity was able to prevent further hippocampal volume atrophy [53]. A 14-year population-based prospective study in Germans [54] found that self-reported regular physical activity was associated with a reduction of risk of developing MCI and AD including enhancements in their neuropsychological test scores. Other prospective studies also reported that total physical activity was associated with reduced risk of developing AD [55]⁠ and significantly lessened rates of cognitive decline [56].

Some RCT studies have shown mixed efficacy of physical activity on cognitive decline, showing positive effects on ADL scores [57, 58] and neuropsychiatric symptoms [59]⁠ but no improvements on behavioural, depressive, or nutritional scores [57]⁠ or cognitive performance [59]. Conversely, a systematic review and meta-analysis of 6 RCTs found that in patients with AD who underwent an exercise program, there were decreased rates of cognitive decline and positives effects on global cognition [60]⁠. Other studies [61, 62]⁠ showed mixed results, making light of methodological concerns (insufficient sample sizes, decrease in compliance, or lack of follow-up), whereby some researchers have rated the quality of evidence to be low, citing that investigating participants with diverse types and severity of dementia would be beneficial in future studies⁠ [63]. A meta-analysis and systematic review by Du and colleagues [64] showed that exercise may improve cognitive function in AD and may potentially decelerate cognitive decline, however this relationship was not always found across studies. Therefore, the requirement for more RCTs with clear intervention criteria, larger sample sizes, and longer-term follow-up intervals are required to further elucidate the benefits of exercise on cognition in AD patients.

### The ALMUTH study and main outcomes of the current study

The ALMUTH study was designed to further explore the potential benefits of music therapy and physical activity with the main aim of decelerating the rate of brain aging in patients with a diagnosis of AD and with secondary aims of improving neurocognitive and neuropsychological well-being. For specific details about the ALMUTH study protocol, see the updated research study protocol [1].

⁠The aim of the randomised pilot trial is to investigate if the inclusion of participants with AD into the ALMUTH protocol is feasible, and if continued recruitment of AD patients is warranted. The current pilot trial was conducted in conformance with the conceptual framework described by Eldridge and colleagues [65] known as the Consolidated Standards of Reporting Trials (CONSORT), in which parts of the future RCT are conducted on a smaller scale to determine the study’s feasibility. Feasibility outcomes were therefore assessed to determine whether the pilot trial was feasible to continue recruitment and testing of AD patients.

## Methods

### Aim

The aim of this study was to examine the feasibility of the ALMUTH study in a group of mild-to-moderate AD patients and to determine if the 12-month intervention RCT protocol is feasible to conduct in such patients.

### Trial design

The study was conducted as a parallel three-arm randomised (1:1:1) controlled trial. The study was conducted in Bergen, Norway, where repeated assessments at baseline, and after 12-month follow-up were completed. The study was also carried out in accordance with the CONSORT Extension to Pilot and Feasibility Trials [65]⁠, with ClinicalTrials.gov identifier NCT03444181.

### Research setting

Neuropsychological testing and neuroimaging were undertaken at Haukeland Hospital and at the Department of Biological and Medical Psychology at the University of Bergen in Norway. Physical training sessions were undertaken at the Western Norway University of Applied Sciences sports facility. MT (singing lessons and twice monthly choir sessions) was delivered by registered music therapists at two locations in Bergen.

### Recruitment

Recruitment was carried out from April 2018 to April 2019. Potential participants were screened over the telephone and then invited for pre-assessment. Recruitment efforts included advertising in newspapers, physical and online brochures, physical flyers distributed in elderly day centres, hospitals, shopping centres, and radio appearances. Efforts included dissemination of flyers and brochures to general practitioners in the Bergen municipality to distribute to patients. Information was shared with outpatient clinics, including the National Association for Public health, Bergen Dementia Association, Polyphonic institute, radio appearances on Norwegian Broadcasting Corporation (NRK), and to elderly day centres in the Bergen municipality, and dementia focused events.

### Participants

Due to safety and ethical requirements, participants were accompanied by a caregiver during the pre- and post-assessment, and throughout the study. Written informed consent was provided by both the patient and caregiver. The caregivers corroborated patient health status, patient medication, and ensured the participants’ continued adherence to the study protocol over the study period. Caregivers were present during the pre- and post-assessment tests and corroborated Instrumental (I-ADL) and Personal (P-ADL) Activities of daily living responses by the patients.

Inclusion criteria involved the recruitment of participants with (1) Norwegian as their first language (2) a medical diagnosis of Alzheimer’s Disease (3) mild to moderate AD as defined by an MMSE score above 10 ([66]⁠ (Moderate AD: between 11 and 20 & Mild AD: between 21 and 30) (4) still living at home (5) able to provide informed consent (6) have an accompanying caregiver present (also providing informed consent), and (7) able to undertake MRI scanning. Participants who moved to a nursing facility during the intervention period were not excluded from the study.

Exclusion criteria for the study were the presence of (1) severe psychiatric disorders (Major Depressive Disorder, Bipolar Disorder, Schizophrenia, Psychotic Symptoms), (2) history of traumatic brain injury (3) neurological disease (e.g., Multiple Sclerosis, Epilepsy) (3) severe auditory impairments (4) physical immobility (5) vascular disorders (history of heart disease, heart attack, heart surgery, stroke), (6) other Dementia types (e.g., Lewy body dementia, Fronto-Temporal Dementia, Vascular Dementia) (7) claustrophobia (8) ferro-magnetic metal in the soft tissue of the body not compatible with MR scanning (e.g., pacemakers) (9) and living in a residential aged care facility.

### Intervention

We recruited Norwegian-speaking mild-to-moderate Alzheimer’s patients for a 12-month RCT. The study included neuropsychological testing and MRI measurements with repeated assessments at baseline, and after 12 months. MRI was performed with three different MR sequences: structural T1-weighted imaging, diffusion tensor imaging, and two resting-state functional MRI acquisitions (one with, and one without a music listening condition, counterbalanced across participants). Subjects were randomised into one of three groups: passive control, CON, (no treatment) and two active non-pharmacological therapy treatments: physical activity, PA, and music therapy, MT (specifically singing lessons). For those randomised into PA or MT, participants were offered up to 40 sessions weekly over 12 months (see Table 1). Intervention details can be found in the updated ALMUTH study protocol [1]⁠.

**Table 1:** Intervention protocol details for weekly treatment sessions during 12-months

Intervention arm	Activity	Session Length (min)	Frequency (/month)	Intensity Level
Physical Activity	Warm-up, individual and group-based PA, cool-down, indoor and outdoor training physical activity, Nordic walking	70–90	4	Low to moderate
Music Therapy	Warm-up, vocal exercises, practice of choir songs, music listening Choir sessions	45–60	4	Low to moderate
Control Group	None	N/A	N/A	N/A

Non-pharmacological therapies in the ALMUTH study by activity type, session length (minutes), frequency of sessions (/month), intensity levels

*N/A* Not Applicable

### Music therapy intervention

Participants randomised to the MT intervention were offered weekly singing lessons by registered music therapists (and music therapy students under the supervision of a registered music therapist). Weekly sessions were designed by the music therapists in collaboration with the researchers. Participants received up to 40 individual 1 on 1 sessions (45–60 min each) of weekly MT over 12 months. Songs were adapted to the participants’ abilities and ranged across varying degrees of difficulty. Participants received recorded study materials (burned cds, and/or MP3 files) which they were instructed to practice 30 min daily. Music therapy sessions included warm-up exercises including breathing techniques, song practice, and passive listening to the participants’ music of choice. Music therapists and students also led twice-monthly choir sessions for participants in the music intervention where participants met in a group setting to perform musical songs taught to them by the music therapists and led by a choir leader. The choir sessions lasted for 45 min. The song difficulty ranged from low to moderate and were adapted to the participants' needs, familiarity, and ability level.

### Physical activity intervention

Participants randomised to the PA intervention were instructed by registered physiotherapists and sports educated personnel with a background in physical activity for adults and seniors. Weekly sessions were designed by the PA group exclusively. For all subjects randomised to the PA group, up to 40 sessions (totaling 70–90 min per session) of weekly group interventions were offered over a 12-month period. Participants received printouts of daily physical activities and were told to practice 30 min daily. Most participants had a friend or caregiver accompanying them during the session. Physical activities were tailored to the intensity level of each participant and taught to their next-of-kin. The intensity levels ranged from low to moderate and were adapted to participants' needs.

### Control group

Participants allocated to the control group were asked to continue their daily routines as per usual and not to attend concurrent research studies during their inclusion. After 12 months, all patients in the CON group were offered to participate free of charge in the MT or PA group sessions and were interviewed on their activity levels during the year.

### Neuropsychological testing and magnetic resonance imaging

The standardised neuropsychological test battery included the following questionnaires: The Consortium to Establish a Registry for Alzheimer’s Disease (CERAD; [67]⁠) World List Memory Test, Lawton Activities of Daily Living (Instrumental and Personal) (I and P-ADL; [68]), Geriatric Depression Scale (GDS; [69]), Mini-Mental State Examination (MMSE-Norwegian Revised version [66, 70]⁠, a computerised version of the Finger Tapping Test (FTT), Stroop Task, specifically the Word Colour Interference Test (CWIT; [71]), stimuli from the Profile of Music Perception Skills (PROMS; [72]) test, and the Short Physical Performance Battery (SPPB; [73]). Changes to the testing protocol were quickly implemented by the researchers to include a replacement of the computerised version of the Stroop task to a paper version of the Delis-Kaplan Executive Function System’s (D-KEFS; [74]⁠) and the online version of the mini-version of the Profile of Music Perception Skills (mini-PROMS; [75]). Neuroimaging was conducted using three measurements: structural MRI (sMRI), diffusion tensor imaging (DTI), and functional MRI (fMRI) with a counterbalanced music listening and non-music listening acquisition. Six instrumental musical genres were selected by the researchers (pop, rock, classical, jazz, world, and folk music) and participants were instructed to select a genre to listen to during the MR recordings. See Table 2 for the order of neuropsychological and neuroimaging assessments and the approximate time duration per test.

**Table 2:** Neuropsychological and neuroimaging testing flow

Tests	Duration (min)
CERAD world list	5–10
I- and P- ADL	7–10
World list delayed recall	5
GDS	5–10
MMSE	7–10
Stroop	5–7
FTT	5
mini-PROMS	20–30
SPPB	5
Neuroimaging	
sMRI	9
DTI	8
fMRI	14

Order of neuropsychological and neuroimaging assessments. Approximate duration per test listed in minutes

### Sample size estimation

Formal sample size estimations are not typically required for pilot trials [76], however, as this pilot trial is part of the larger study intended solely for the inclusion of AD patients, the sample size was estimated. Sample size estimation was conducted using G* Power (G*Power 3), to reach a medium effect size (between Cohen’s d = 0.5 and 0.8), an estimate of 35 participants per group was sufficient to reach 80% statistical power with a two-tailed significance level of 2.5%. To account for expected attrition rates of 22.22%, estimates of 45 participants per group for a total of 135 study participants across three groups were calculated. The aim was to recruit 135 participants over the first 12 months.

### Randomisation, sequence generation, and allocation concealment

Randomisation was conducted after participants were screened for eligibility, consent forms were signed, and MRI and neuropsychological tests were completed. The randomisation procedure (1:1:1) was done via computer generated randomisation sequence using the software R and concealed by an external researcher not directly involved with the participants. Block randomisation with randomly varying block sizes of 3 or 6 were used to ensure balance and unpredictability. After participant baseline tests and MR recordings were completed, randomisation results were sent by email to the researchers who then contacted the participants by phone and allocated them to one of three groups: MT, PA, or CON.

### Blinding

The ALMUTH study is an open label study and therefore both researchers and participants were aware of group allocation after randomisation.

### Statistical analysis

Descriptive statistics were used to compare the three allocated groups. The study was conducted with the intention-to-treat principle [77], which means all participants were analysed in the group to which they were randomised, regardless of whether they received the allocated intervention. All analyses were undertaken using SPSS version 26.0.0.0. (IBM, Armonk, New York, U.S.A.).

### Feasibility outcome measures

The study would be deemed feasible if the study met the criteria found in Table 3.

**Table 3:** Feasibility outcome measures and target values

Target Values
1. Recruitment rate over 11.25 subjects per month over the course of 12 months
2. Recruitment target of 135 participants reached over the course of 12 months
3. Recruitment costs approximately 100 EUR per subject
4. Attrition rates less than 25% based on sample size calculations
5. Differential attrition rates no less than 25% per study arm
6. Retention rates over 75%
7. Adherence/Compliance to protocol with at least 80% of attendance to weekly sessions
8. Location, room sizes, and equipment were sufficient
9. Completeness of data at least 90% complete for baseline and follow-up tests
10. Acceptability of intervention by study personnel and minimal issues raised by staff
11. Study fidelity adherence by staff and researchers

Listed are the 11 target values for feasibility of ALMUTH study

## Results

### Participants

**Fig. 1:**
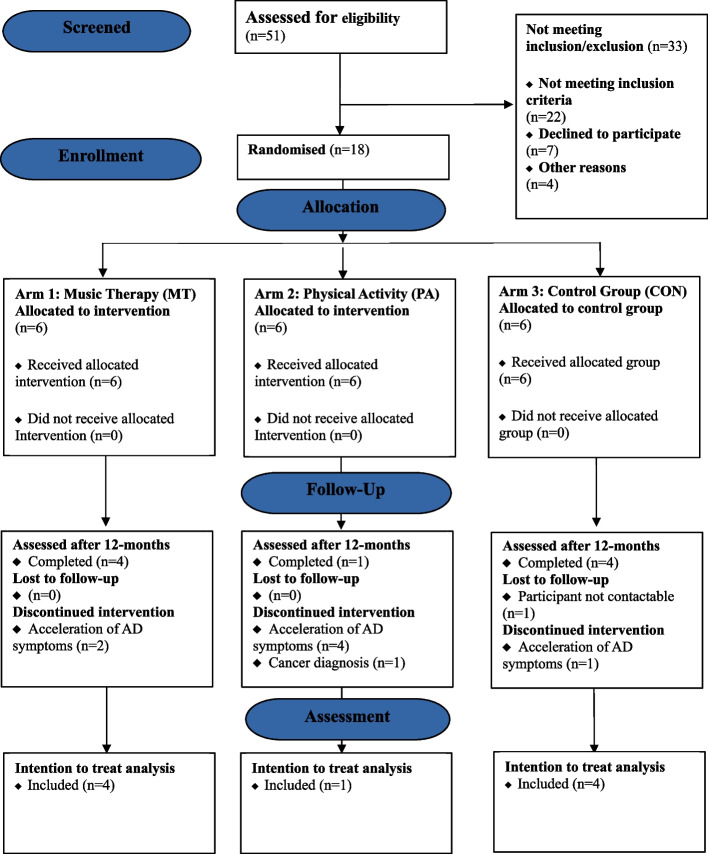
CONSORT diagram showing flow-through study for participants assessed for eligibility

Fifty-one participants were assessed for eligibility of which eighteen right-handed Norwegian AD patients (10 Male and 8 Female) with a mean age of 74.89 years (*SD* = 6.56) were randomised between April 2018 and April 2020. The participants had no previous ailments and had acquired an AD diagnosis by a physician. Demographic details at baseline can be found in Table 4 and a detailed flow chart detailing recruitment and participant flow can be found in Fig. 1.

**Table 4:** Demographic characteristics at baseline assessment

Baseline Characteristics	Control Group n/N (%), (*N* = 6)	Physical Activity n/N (%), (*N* = 6)	Music Therapy n/N (%), (*N* = 6)
**Demographics**			
Age (mean (*SD*))	76.67 (8.38)	73.83 (5.49)	77.17 (6.05)
Gender			
Male	3/6 (50)	3/6 (50)	4/6 (66.67)
Female	3/6 (50)	3/6 (50)	2/6 (33.33)
Handedness			
Right	6/6 (100)	6/6 (100)	6/6 (100)
Left	0/6 (0)	0/6 (0)	0/6 (0)
Both	0/6 (0)	0/6 (0)	0/6 (0)
Years of Education (mean (*SD*))	13.33 (2.16)	17.17 (3.49)	13.17 (3.19)
**Diagnosis based on MMSE cutoff**			
Mild AD	5/6 (83.33)	4/6 (66.67)	5/6 (83.33)
Moderate AD	1/6 (16.67)	2/6 (33.33)	1/6 (16.67)
**Self-Reported Medical History**			
Hearing Deficit	2/6 (33.33)	1/6 (16.67)	3/6 (50)
Medication type			
AD Medication	5/6 (83.33)	5/6 (83.33)	3/6 (50)
Blood Pressure	1/6 (16.67)	2/6 (33.33)	3/6 (50)
Anxiety	0/6 (0)	0/6 (0)	1/6 (16.67)
Sleeping	0/6 (0)	0/6 (0)	0/6 (0)
Cholesterol	2/6 (33.33)	0/6 (0)	2/6 (33.33)
Allergy	2/6 (33.33)	0/6 (0)	0/6 (0)
Metabolism	0/6 (0)	1/6 (16.67)	1/6 (16.67)
Anti-depressants	2/6 (33.33)	1/6 (16.67)	0/6 (0)
Previous Ailments	0/6 (0)	0/6 (0)	0/6 (0)
**Lifestyle characteristics and background**			
Smoking^a^	1/5 (20)	1/4 (25)	4/4 (100)
Singing alone	4/6 (66.67)	4/6 (66.67)	5/6 (83.33)
Singing in public	4/6 (66.67)	4/6 (66.67)	2/6 (33.33)
Dance	3/6 (50)	4/6 (66.67)	4/6 (66.67)
Currently physically active	2/6 (33.33)	6/6 (100)	4/6 (66.67)
Grew up with music in the home	4/6 (66.67)	6/6 (100)	5/6 (83.33)
Parents sung lullabies during childhood	4/6 (66.67)	6/6 (100)	3/6 (50)
Have pets	0/6 (0)	0/6 (0)	1/6 (16.67)

Demographic descriptive statistics at baseline

*M* Mean, *SD* Standard Deviation, *n* sample number

^a^Missing Data

### Consent, Intention-to-Treat, and Harms

No issues were raised regarding consent by the participants and their caregivers. Consent was achieved by 100% of the patients and their caregivers. No participant randomised to a group deviated from their original group allocation. No adverse events were reported from the participants and/or caregivers.

### Time requirements for recruitment and interventions

Recruitment and testing periods were set to be conducted between April 2018 and April 2020. Recruitment began between April 2018 and April 2019, while intervention sessions occurred over 12 months after the initial pre-assessment date. The study sample size requirement of 135 AD participants was not reached during the initial recruitment period (April 2018 and April 2019). As the PA intervention began 8 months after the MT intervention, data was irregular and incomplete. Contractual PA agreements and organisational agreements were not finalised, delaying the timeline of the PA intervention start. This meant that of the six randomised individuals in the PA group, three of them were unable to attend any treatments and subsequently dropped out of the study.

### Feasibility outcomes

#### Recruitment* rate*

The recruitment of AD patients was conducted between April 2018 and April 2019. Eighteen eligible participants were randomised from a total of 51 telephone screened potential participants resulting in 35.29% of potential participants being randomised and booked for baseline assessments. The target goal was to recruit at least 11 subjects per month, yet the actual value was approximately 1.5 subjects recruitment per month.

#### Recruitment target achieved

Recruitment of eligible, telephone screened, and randomised participants yielded 18/135 (13.33%) of the target goal of 135 patients over the course of 12-months.

#### Recruitment* costs*

The initial advertising budget was 14,000 EUR, which accounted for approximately 100 EUR per 135 total projected participants. Recruiting costs for 18 participants cost 9,000 EUR, which resulted in 500 EUR costs per participant, equivalent to five times the budgeted amount per individual.

#### Retention* rates*

Target retention rates were over 75% retention of participants. Actual retention rates were 50% where 9/18 participants continued the intervention to completion with follow-up neuroimaging and neuropsychological tests 12-months later.

#### Attrition (Dropout)* rates*

To account for attrition, estimates of 45 participants per group for a total of 135 study participants across three groups was calculated, accounting for about 22.22% total attrition rates (< 25%). Actual attrition rates were 50%, where 9/18 participants dropped-out due to the acceleration of AD symptoms, difficulty to contact participant, relocation to nursing homes, family members unable to continue due to the difficulty of the requirements of the study, and/or developed other illnesses or ailments like getting a cancer diagnosis or breaking a hip. To ensure transparent reporting [78, 79], reasons for withdrawal can be found in Table 5.

**Table 5:** Reasons for withdrawal from the ALMUTH study

Reasons for Withdrawal	n/N, *N* = 9
Accelerating AD symptoms and AD progression	7/9
Caregiver unable to continue	5/9
Lived far away and unable to attend sessions regularly	2/9
Other illnesses (cancer diagnosis and broken hip)	2/9
Dissatisfied with the PA intervention	3/9
Unable to contact participant for follow-up test	1/9

Of the nine participants who withdrew, several reasons were given, and the table shows how many complaints were made from a total of the nine participant dropouts by the patients and / or caregivers

#### Differential attrition rates by study arm

To account for differential attrition rates, estimates of 45 participants per group for a total of 135 study participants across three groups was calculated (10 dropouts per group), accounting for about 22.22% attrition rates (< 25%). The differential attrition rate of the MT group was 2/6 (33.33%), followed by the PA group at 5/6 (83.33%), and the CON group 2/6 (33.33%). The PA group had more dropouts compared to the MT and CON groups. PA dropouts were mostly due to the PA intervention researchers not ready to begin the intervention until February 2019. Lateness to start the intervention in combination with accelerating AD symptoms contributed to the large dropout rates witnessed in the PA group.

#### Adherence to protocol: attendance rates

Of the nine who completed the protocol, four of the nine were in the CON group. The one participant in the PA group attended 19 sessions, approximately 47.5% of the total offered sessions (up to 40 sessions total). The four MT participants joined anywhere from 8, 15, 21, and 40 sessions, which is equivalent to the participants joining approximately 52.5% of their total sessions during a year. Both the MT and PA groups combined allowed for a 50% global attendance to protocol. Adherence rates were set at 80% attendance to be included in the main analyses, however only one participant out of the five who completed an intervention had 100% adherence to the protocol.

### Control participant activities during 12-months

CON group patients were instructed not to participate in any other studies over the ALMUTH study and to continue with their regular routines over the year. Participants were questioned during post-assessment about their activity levels during the year. Of the four CON participants who completed the ALMUTH protocol, one participant admitted to joining a three-month music therapy project. Three of the four did not deviate from their typical routine.

#### Adequate equipment: location, room size, and equipment

The physical activity intervention was located at the Western Norway University of Applied Sciences sports facility. The facility was equipped for individual and group-based indoor and outdoor athletics, sports, and hiking. As for the music therapy intervention, music therapists acquired two rooms for the singing lessons to occur with optimal space to hold a choir ensemble. Location, room size, and equipment were deemed sufficient across the intervention groups.

#### Data completeness

The participants who completed pre- and post-neuropsychological assessments had a data completion rate of 95.56%. In terms of neuroimaging scans, the scanning time per participant was reduced to the lowest possible scanning time. Most of the participants were able to be scanned pre- and post- with the exception of two participants who could not be scanned during the first pre-test (e.g., improperly screened for metal in the body, movement, forgetting why they were in the scanner, and agitation). One of the two participants was able to be screened during the post-test assessment.

#### Acceptability of intervention by staff

### Issues faced by the physiotherapists and sports scientists

In the physical activity group sessions, the sports scientists and physiotherapists found that the AD participants faced challenges in being able to move or cooperate with other participants without one or two students assisting them, and challenges connected to pharmaceutical side effects as rigidity and start/stop difficulties (e.g., parkinsonism). The main problem for a group-based intervention was the rapidly declining health of the participants, and the increased demand on the staff to run sessions (e.g., one or two helpers per participant). This affected group cohesion and did not allow for the creation of a sense of community between the participants, which also further limited the implementation of co-operative activities and/or games. Due to the heterogeneity of the participants, along with their rapidly declining health and functional status, it became difficult to meet individual demands and maintain group activities. Activity and intensity levels decreased, which can affect potential physical outcomes (e.g., coordination, strength, endurance).

### Issues faced by caregivers

In terms of additional operational issues, the physical therapy activities were conducted each wednesday in the early evening at a gymnasium at the Western Norway University of Applied Sciences. Participants had to travel each week to the site. The PA sessions are dependent on their caregivers for transportation to the intervention location. Several caregivers struggled getting the participants to the sessions (e.g., getting dressed and ready, moving out the door, getting into the car, driving to the facility, getting the patient out of the car and into the building). This struggle was repeated on the way home, and for an intervention of a 12-month long duration, this became burdensome on the caregivers. Some of the dropouts were due to caregivers unable to meet the demands of the protocol.

### Issues faced by the musical therapists

There were fewer issues to contend within the musical therapy sessions as each session was individualised and based on a more flexible appointment schedule that suited the participant (as opposed to the PA group that met at a fixed time due to gymnasium availability). The musical therapists in some cases went to the homes of the participants who were unable to meet at the physical location music therapy sessions were held. Twice-monthly choir sessions were held, however, some spouses did not have time to bring the participants each time. Some caregivers did not enjoy transporting the participants to the choir sessions.

#### Study fidelity: adherence by researchers and staff

Incomplete or missing subscale data and poorly administered tests accounted for 28.89% of missing baseline data (SPPB, CERAD word list, MMSE, CWIT, and PROMS). Further, most participants had difficulty with the computerised version of the Stroop task. The subsequent use of a paper version of the D-KEFS⁠ was quickly implemented and well-tolerated. Incorrectly coded stimuli taken from the PROMS was also replaced by the online validated version of the mini-PROMS [75].

Coordination for multi-site procedures was difficult to fully implement. Ideally, all collaborators would prepared to deal with AD participants and have the necessary infrastructure ready in advance. The music therapists were prepared and were consistent with intervention leading to more overall sessions completed by the music group. Due to insufficient funding and unforeseen planning difficulties, the physical activity was late to begin their interventions beginning eight months later than the MT sessions began (February 20, 2019). Both intervention partners and researchers were consistent throughout the study, keeping to the protocol closely, and were sensitive to the needs of the patients and their caregivers.

Lastly, the staff included three music therapists, three sports trained personnel, and multiple physical education students. Research staff included two doctoral candidates, and rotating research assistants, along with supervision from a post-doctoral researcher and the principal investigator. Ideally, it would have been prudent to hire a full research manager to administer the recruitment, advertising, and project administration along with additional research technicians. Feasibility outcomes including target and actual outcomes can be found in Table 6.

**Table 6:** Table of feasibility outcomes including target and actual outcomes

Feasibility Outcome	Target outcome	Actual outcome
1. Recruitment rate	> 11.25 subjects/month	1.5 subjects/month
2. Recruitment total	135 subjects	18 subject
3. Recruitment costs	100 EUR per subject	500 EUR per subject
4. Retention rates	> 75%	50%
5. Attrition rates	< 25%	50%
6. Different attrition rates per study arm:		
I. MT	< 25%	2/6 (33.33%)
II. PA	< 25%	5/6 (83.33%)
III. CON	< 25%	2/6 (33.33%)
7. Adherence to protocol	80% attendance	50%
8. Adequate equipment	Room, Location, and Equipment was adequate	Room, Location, and Equipment was adequate
9. Data completeness	> 90% Completeness	95.56%
10. Acceptability of intervention by staff	No issues raised by staff	Many issues raised by staff
11. Study fidelity adherence by staff and researchers	No study fidelity issues	Presence of study fidelity issues

Key feasibility assessments of target and actual outcomes reached

## Discussion

As the pathophysiological process of AD has been shown to begin years before the onset of clinical symptoms [3, 80], and due to established pathological burden the focus on developing safe and effective interventions in early and presymptomatic AD stages has been encouraged. The importance of performing pilot and feasibility studies not only informs larger trials [81] but is also used to test procedures that can be applied to the main study [82], and to provide useful information across various processes required to implement such a trial⁠. The randomised controlled pilot trial was conducted as a retrospective feasibility assessment of the ALMUTH study to optimise the quality of the trial and determine if the strict inclusion of mild-to-moderate AD patients was feasible. Assessments of the feasibility criteria found that the pilot trial was not feasible exclusively in AD patients, and that the expansion into prodromal and preclinical stages is recommended.

### Limitations

In terms of the fidelity of the study, cooperation with research partners is both a limitation and a necessity. Good working partnerships and timely cooperation can expand research and knowledge, but improperly synchronised teams can create many difficulties for the management of such a large-scale project. Inappropriate test use was quickly rectified by the current researchers on the team and higher quality tests were swiftly implemented (mini-PROMS and D-KEFS). For a large study of this duration, it would be necessary to ensure that enough personnel are hired, tasks equally divided, and budget costs allocated for replacement of staff if needed.

Due to unforeseen economic and planning needs, the PA partners were unable to begin offering sessions until February 2019, 8 months after the MT and CON groups began. This is a significant limitation to the current study and has been since rectified in the expanded and upgraded version of the ALMUTH study protocol [1]⁠. The study’s length also imposed considerable restrictions on the participants’ adherence to the program and the caregivers’ ability to follow-up weekly implementation over the study period.

Expensive advertising and recruitment costs, in addition to ancillary expenses (due to the needs of the PA group) revealed that it may be important to budget additional funds and that future studies wishing to implement similar large-scale interventions in dementia patients should be wary of cost inefficiencies as well as recruitment difficulties, attrition rates, and retention rates, specifically in a small city like Bergen, Norway with 300,000 inhabitants. In addition, the presence of competing research and university institutions simultaneously conducting studies on AD contributed to excess competition for access to the small sample pool of AD patients in Bergen. Furthermore, our research group did not have access to a hospital registry which other institutions had access to, making recruitment efforts more arduous and the discovery of available and eligible AD potential participants more difficult. In sum, future studies should be made aware of recruitment difficulties, poor participant retention and attrition rates, and concerns about follow-up assessments with patients who have progressed in their AD diagnosis.

### Generalisability

In preparation for similar future RCT studies assessing Alzheimer’s Disease patients, the current randomised pilot trial may offer suggestions for further improvements and conduct. As the RCT design is considered the gold standard for effectiveness in research [83], the study can include milder forms of memory impairments such as Mild Cognitive Impairment and Subjective Cognitive Decline, whereby the participants are healthier and may be able to adhere and better comply to the protocol of the study. Those in preclinical stages may not be as dependent on caregivers, and attrition and retention rates may improve.

Empirical evidence suggests that regardless of intervention type, participants who adhere to the study until the end of the protocol tend to fair better than those who do not [82]⁠. Future studies may want to include a shorter intervention period (6 months in duration) or be aware of the difficulty in compliance and stamina required for such a longitudinal study. As each participant must join up to 40 sessions during a one-year interval, the compliance rates to joining sessions were low. Thus, low adherence and compliance to the study protocol can inform future studies not to be overly optimistic in their ‘recruitment to completion’ estimates.

### Interpretation

According to Thabane and colleagues [76], there are four outcomes of a pilot study which can be found in Table 7.

**Table 7:** Interpretation of pilot study outcomes

Outcomes
1. Stop: a main study is not feasible
2. Continue with study protocol modifications: main study is feasible but will require further modifications
3. Continue without modifications, but monitor carefully: main study is feasible but requires close attention
4. Continue without modifications: a main study is feasible and there is no further requirement to modify the protocol

Four outcomes of a pilot study

Reviewing the feasibility outcomes and based on the options in Table 7, the randomised pilot trial falls under category 1 (Stop: a main study is not feasible). Reviewing the feasibility of such a longitudinal study in early phase AD patients, it is not recommended to further recruit AD patients for the ALMUTH study or focus solely on AD recruitment in general.

### Current amendments to the ALMUTH study

Taking account of the issues raised in the pilot trial, the ALMUTH study is currently recruiting subjects in preclinical and prodromal stages, such as participants with Mild Cognitive Impairment (MCI) (as diagnosed by a physician), Subjective Cognitive Decline (as categorised by the use of the Subjective Cognitive Decline Questionnaire (SCD-Q; [84]⁠, whereby both MyCog and TheirCog SCD-Q Scores are above 7), and participants claiming declining memory and who do not meet the criteria of the SCD-Q (MyCog and TheirCog scores under 7) or are diagnosed with prodromal dementia. The ALMUTH study has also introduced an additional memory test which has been suggested for use by the International Working Group called the Free and Cued Selective Reminding Test (FCSRT; ⁠[85]). The FCSRT includes 16 pictorial stimuli which considers Immediate Recall, Free Recall, and Delayed Recall (also called pFCSRT + IR). Additional upgrades to the study include the inclusion of the revised Norwegian Dispositional Resilience Scale 15 items (DRS-15; [86]), the self-reported musical skills questionnaire (using two subscales: active engagement and musical training of the Goldsmiths Musical Sophistication Index (Gold-MSI; [87]), and the addition of physical ability measures and caregiver assessments which can be found in detail in the revised study protocol [1]. ⁠

## Conclusion

Feasibility of the above randomised controlled pilot trial revealed that the ALMUTH protocol is not feasible exclusively in mild-to-moderate AD patients. Taking into account the feasibility issues addressed in this pilot trial, the ALMUTH study protocol has been extended to include individuals in prodromal and preclinical stages.

## Data Availability

Data and materials are available upon request.

## Abbreviations

ALMUTH: Alzheimer’s and Music Therapy Study
AD: Alzheimer’s Disease
CWIT: Colour Word Inteference Test
CERAD: Consortium to Establish a Registry for Alzheimer’s Disease
CON: Control group
D-KEFS: Delis-Kaplan Executive Function Systems
DTI: Diffusion Tensor Imaging
DRS: Dispositional Resilience Scale
FCSRT: Free and Cued Selective Reminding Task
FFT: Finger Tapping Test
fMRI: Functional Magnetic Resonance Imaging
Gold-MSI: Goldsmiths Musical Sophistication Index
MRI: Magnetic Resonance Imaging
MCI: Mild Cognitive Impairment
mini-PROMS: Mini-version of the Profile of Music Perception Skills
MT: Music Therapy
NPTs: Non-Pharmacological Therapies
PA: Physical Activity
RCT: Randomised Controlled Trial
SPPB: Short Physical Performance Battery
sMRI: Structural Magnetic Resonance Imaging
SCD: Subjective Cognitive Decline
